# An observational cohort study of the performance of the REDS score compared to the SIRS criteria, NEWS2, CURB65, SOFA, MEDS and PIRO scores to risk-stratify emergency department suspected sepsis

**DOI:** 10.1080/07853890.2021.1992495

**Published:** 2021-10-22

**Authors:** Narani Sivayoham, Adil N. Hussain, Luke Shabbo, Dylon Christie

**Affiliations:** Department of Emergency Medicine, St George’s University Hospitals NHS Foundation Trust, London, UK

**Keywords:** Clinical prediction rule, emergency department, prognosis, discrimination, sepsis, septic shock

## Abstract

**Objective:**

To compare the performance of the Risk-stratification of Emergency Department suspected Sepsis (REDS) score to the SIRS criteria, NEWS2, CURB65, SOFA, MEDS and PIRO scores, to risk-stratify Emergency Department (ED) suspected sepsis patients for mortality.

**Method:**

A retrospective observational cohort study of prospectively collected data. Adult patients admitted from the ED after receiving intravenous antibiotics for suspected sepsis in the year 2020, were studied. Patients with COVID-19 were excluded. The scores stated above were calculated for each patient. Receiver operator characteristics (ROC) curves were constructed for each score for the primary outcome measure, all-cause in-hospital mortality. The area under the ROC (AUROC) curves and cut-off points were identified by the statistical software. Scores above the cut-off point were deemed high-risk. The test characteristics of the high-risk groups were calculated. Comparisons were based on the AUROC curve and sensitivity for mortality of the high-risk groups. Previously published cut-off points were also studied. Calibration was also studied.

**Results:**

Of the 2594 patients studied, 332 (12.8%) died. The AUROC curve for the REDS score 0.73 (95% confidence interval [CI] 0.72–0.75) was significantly greater than the AUROC curve for the SIRS criteria 0.51 (95% CI 0.49–0.53), *p* < .0001 and the NEWS2 score 0.69 (95% CI 0.67–0.70), *p* = .005, and similar to all other scores studied. Sensitivity for mortality at the respective cut-off points identified (REDS ≥3, NEWS2 ≥ 8, CURB65 ≥ 3, SOFA ≥3, MEDS ≥10 and PIRO ≥10) was greatest for the REDS score at 80.1% (95% CI 75.4–84.3) and significantly greater than the other scores. The sensitivity for mortality for an increase of two points from baseline in the SOFA score was 63% (95% CI 57.5–68.2).

**Conclusions:**

In this single centre study, the REDS score had either a greater AUROC curve or sensitivity for mortality compared to the comparator scores, at the respective cut-off points identified.

**KEY MESSAGES**
- The REDS score is a simple and objective scoring system to risk-stratify for mortality in emergency department (MED) patients with suspected sepsis.
- The REDS score is better or equivalent to existing scoring systems in its discrimination for mortality.

## Introduction

Sepsis, a life-threatening condition [1] is a frequent finding in hospitalized patients [2]. Hospitalized patients with sepsis are known to have higher mortality rates and longer lengths of stay compared to hospitalized patients with non-sepsis related diagnoses [2]. Several studies have found that the majority of hospitalized patients with sepsis are admitted as emergencies [3–5]. Early identification and management of patients with sepsis are advocated [6,7]. This gives the emergency department (ED) a pivotal role in the identification and the initiation of treatment. Early identification can be facilitated by the use of a highly sensitive screening tool. This will identify patients who are likely to have sepsis and facilitate further tests and treatments, whilst in the ED. The management of the patient with suspected sepsis should not simply stop there. Patients with suspected sepsis should then be risk-stratified so that the sickest patients who are at the highest risk of death are identified in the ED. This would enable further focussed care to be implemented to improve outcome. Such risk-stratification requires an objective tool such as a scoring system.

We have previously derived and validated the Risk-stratification of Emergency Department suspected Sepsis (REDS) [8] score by combining the qSOFA [9] and the simplified-mortality In Severe Sepsis in the ED (sMISSED) [10,11] scores with refractory hypotension (RH) and initial lactate [12,13]. The REDS score has eight variables; six dichotomous and two stratified variables. The dichotomous variables are as follows: Initial respiratory rate (RR) ≥22 breaths per minute, initial systolic blood pressure (SBP) ≤100 mmHg, altered mental state, age ≥65 years, serum albumin ≤27 g/l and International normalized ratio (INR) ≥1.3. When any of the six dichotomous variables are present, they each score 1 point (maximum total score of 6 points). The stratified variables are RH (maximum score 3) and initial lactate (maximum score 3). The absence of RH scores 0 points. When RH (the requirement for vasopressors after 30 ml/kg of fluid to maintain MAP >65 mmHg) is present and the lactate measurement taken after the fluid bolus is ≤2mmol/l, the score is 2 points; and if the lactate is >2 mmol/l the score is 3 points. The initial lactate is scored as follows: ≤2 mmol/l scores 0, 2.1–3.9 mmol/l scores 1 point and a lactate ≥4 mmol/l scores 3 points. The REDS score ranges from 0 to 12 with a cut-off point of 3. A REDS score of ≥3 places the patient in the high-risk category for mortality. When all variables are measured, scores of ≥3 has an odds ratio for mortality of over 10 compared to scores of 0–2 [8]. Due to the requirement for blood results and evaluation of the response of the blood pressure to a 30 ml/kg fluid bolus, the REDS score can only be calculated a couple of hours after arrival in the ED.

The REDS score is not the first risk-stratification tool to be used to categorize patients with suspected sepsis in the ED. Other scoring systems, which we will collectively refer to as comparator scores, have been derived and validated or advocated for use for this purpose.

First, the Systemic Inflammatory Response Syndrome (SIRS) criteria [14]. The presence of two of the four variables was used to define sepsis [14], in the presence of infection. However, recent studies have found that the SIRS criteria are not predictive of mortality [15–17].

Second, the National Early Warning Score 2 [18] is recommended by the Royal College of Physicians of the United Kingdom (London, UK) for monitoring patients who are admitted to hospital. It is based on the six vital signs of a patient and the use of oxygen. Although it is recommended for use to risk-stratify patients with sepsis [18–21], it is not a sepsis-specific score.

Third, the “Confusion, Urea, RR, Blood pressure and age of 65 years and above” (CURB65) score [22] is a relatively simple score with only five dichotomous variables. Although the CURB65 score was derived and validated in the context of pneumonia, it has been found to be predictive of mortality in general sepsis in the ED [23].

Fourth, the sequential organ failure assessment (SOFA) score [24], is used to define sepsis in the Sepsis-3 [1] definition. An increase of two points in the SOFA score from baseline is associated with a mortality rate of over 10% and forms the current definition of sepsis. It is the cumulative value of six organ systems scored from 0 to 4. But there is little evidence that the SOFA score is actively used in the clinical setting in the ED as it is complex and unfamiliar to ED clinicians.

Fifth, the Mortality in Emergency Department Sepsis (MEDS) score [25], probably the most widely studied, was derived and validated in 2003. The MEDS score is made up of nine variables but is not entirely objective. It awards the highest score of 6 points to the subjective opinion of the physician regarding the likelihood of death within 30 d. Furthermore, the MEDS score uses nursing home residency as a risk factor for mortality. In our view, the purpose of a risk-stratification tool is to identify patients who are at high-risk of mortality to allow increased and aggressive treatments to be implemented. Incorporating nursing home residency in the score as a risk factor for mortality, a group of patients in whom aggressive treatments are less likely to be implemented, reduces the usefulness of the score.

Sixth, the ED specific “Predisposition, Infection, Response, Organ failure” (PIRO) score was derived and validated in 2011 [26]. It is made up of 15 variables which make it onerous to use in a pressured environment like the ED. Like the MEDS score, it includes nursing home residency. For reasons stated above, we find this reduces the usefulness of the score.

As there are several established scoring systems, each with its own challenges, it is important to know if the REDS score provides any advantage. We hypothesize that the REDS score is equivalent to the comparator scores. Performance of scoring systems is assessed by comparing their discrimination (the area under the receiver operator characteristic [AUROC] curve and the test characteristics) and calibration (Hosmer–Lemeshow [27] “goodness-of-fit” test) [28] for outcome.

The aim of this study is to compare REDS score to the six comparator scores based on the following criteria: (i) the AUROC curve and (ii) the sensitivity for mortality of the high-risk category and (iii) calibration of the scores. The primary outcome measure was in-hospital all-cause mortality.

## Methods

### Setting, study design and population

This study was carried out in the ED of a university teaching hospital in London, UK. The department sees approximately 130,000 adult patients annually. This is a retrospective study of data in the ED suspected sepsis database, a convenience sample. Entry criteria to the ED suspected sepsis database is as follows: Adult patients who received intravenous antibiotics for the treatment of suspected sepsis in the ED prior to admission and a minimum 3 points on the NEWS2 score on arrival or have a SBP <100 mmHg on arrival or have suspected neutropenic sepsis – having had chemotherapy in the preceding 6 weeks or other conditions that may cause bone marrow suppression (e.g. aplastic anaemia and drug-induced agranulocytosis). Patients who received intravenous antibiotics for prophylaxis were not included. Examples of this latter group would be patients who received antibiotics for an open fracture, those who received antibiotics where the primary diagnosis was a gastrointestinal haemorrhage or a convulsion that was not precipitated by an infection. The decision to include or exclude these patients was based on the contemporaneous notes made by the clinician. Patients presenting with a primary diagnosis of COVID-19 were excluded as they can be diagnosed in the ED with point-of-care tests and stratified using more specific scoring systems that have been developed.

### Study period and data collection

Adult patients who were admitted between 1 January and 31 December 2020 who satisfied the above criteria were studied. The patient’s initial vital signs, the WCC, platelet count, INR, the use of warfarin or a direct oral anticoagulant (DOAC), urea, creatinine, bilirubin, serum albumin, C-reactive protein, lactate, presence of RH, COPD and other comorbidities, such as dementia, malignancy, the ability to live independently, use of long-term oxygen therapy (LTOT) and previous Do-Not-Attempt-Resuscitation (DNAR) orders (community or in-hospital), are routinely collected. The ability to live independently was measured as follows: Patients requiring a minimum three-times-a-day care package, where a carer visits the patient three times a day to attend to the patient’s needs, up to nursing home residency were grouped together. This was done as we were aware that many patients who were completely dependent on carers for their activities of daily living, lived at home with either a care package or a carer. A three-times-a-day care package seemed a reasonable indicator of dependency as the patient would be dependent on a carer for activities of daily living. Additional data on the presence of liver disease and the spread of any malignancy were collected. Patients’ baseline blood results were noted for the calculation of the baseline SOFA score. The clinician’s subjective opinion on whether the patient may die in 30 d or terminal illness was judged from the contemporaneous clinical notes. The patients were followed up to discharge from the hospital and their outcome at discharge, the final diagnosis of infection or not and the source of infection were retrieved from the clinical notes and discharge letters. The method of arrival, admission to the intensive care unit (ICU) and hospital length of stay were also noted.

The ED notes of those patients who received intravenous antibiotics were reviewed by researchers (the authors) who are doctors. The data on patients meeting the inclusion criteria were collected for the purpose of continuous audit. The researchers were trained to extract the data from the contemporaneous clinical notes. The data was entered on to an electronic spreadsheet (Excel). All data outside the normal range were rechecked by a second researcher against the original data and corrected where necessary, prior to being anonymized.

The REDS, SIRS, NEWS 2, CURB65, SOFA, MEDS and PIRO scores were calculated retrospectively using the initial vital signs, blood tests and the required co-morbidities. Receiver operator characteristic (ROC) curves were constructed for the REDS and comparator scores. The cut-off points were identified for each scoring system by the statistical software program. Patients who had scores above the cut-off point were deemed to be in the high-risk population. The test characteristics were calculated for the high-risk populations. The sensitivities for mortality and the AUROC curves were compared. The significance of the calibration of the score was also noted.

The test characteristics of the scores were also re-calculated for other cut-off points that are advocated in the literature. With regard to the NEWS2 score, several different cut-off points have been advocated for the purpose of screening for sepsis: Keep et al. [29] advocate using an aggregate score of ≥3; Corfield et al. [30] suggest a score of ≥5; the UK Sepsis Trust [19] (endorsed by the Royal College of Emergency Medicine, London, UK) recommend the use of a NEWS2 score of 3 in one variable amongst other “Red Flag” criteria. The Royal College of Physicians use a NEWS2 score of ≥7 to categorize high-risk.

The test characteristics for a cut-off point of ≥8 [25] for the MEDS score and ≥10 for the PIRO score [26] were also calculated, if they were different to the cut-off points identified by the software package.

As the percentage of bands is not readily available in the UK, this variable was not scored in the MEDS or PIRO scores. The MEDS score was recalculated after excluding the subjective opinion of the clinician on the predicted outcome at discharge and the AUROC curves were compared.

Arterial blood gas results were not available for the majority of patients in the ED. We therefore used the SaO_2_ (peripheral oxygen saturation)/FiO_2_ ratio [31] for the respiratory component of the SOFA score. With regard to the mean arterial pressure (MAP), a score of 1 point was allocated if the initial MAP or the MAP after a fluid bolus was <70 mmHg and a score of 3 points if RH was present. The baseline SOFA score and the admission SOFA scores were calculated. The change in SOFA (ΔSOFA) score was also calculated. The test characteristics of a minimum increase of two points in the SOFA score from baseline were also calculated.

The REDS score was divided into bands of 0–2, 3–4, 5–6 and ≥7. The percentage distribution of the different band of the REDS score in the study population and the deceased population, were also studied. Mortality rates for the different bands of REDS scores were calculated and the mortality rates, the ROC curve and the cut-off point were recalculated after exclusion of those with previous DNAR orders.

### Sample size

The minimum sample size required to perform this study would be a population with 10 deaths per variable in the score [32]. The PIRO score had the largest number of variables at 15 variables. Thus, the population studied should have a minimum 150 deaths.

### Missing variables

Missing variables (blood results) were scored 0 when calculating any of the scores. This decision was made based on the practical use of the scores. This does not imply that the missing variable was normal. For patients on warfarin or a DOAC, a score of 0 was allocated for INR, in the REDS score. The AUROC curves of the REDS score and the comparator scores were compared again after excluding patients with missing values for lactate, albumin and INR.

### Statistics

MedCalc Statistical Software version 19.7 (MedCalc Software Ltd, Ostend, Belgium) was used for statistical analysis. The baseline variables were checked for normality. If normally distributed, they were described as mean with standard deviation. When normality was rejected, the data was described as a median together with its interquartile range. Where normality was rejected for continuous variables, univariate analysis was carried out using the Mann–Whitney test. Differences in categorical variables were analysed using the chi-square test. Statistical significance was defined as *p* < .05. The difference in AUROC curves was assessed by the DeLong method [33] and calibration (goodness-of-fit) was assessed by the Hosmer–Lemeshow test [27] where a *p* value of >.05 signifies satisfactory calibration.

## Results

**Figure 1.**
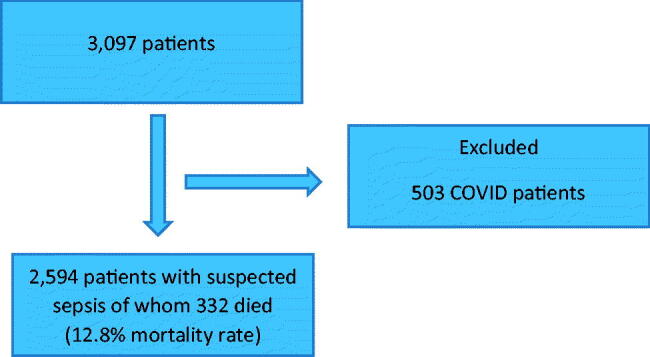
Patient flow diagram.

Of the 3097 patients in the sepsis database, 503 patients with COVID-19 were excluded (Figure 1). Of the 2594 patients studied, 332 died in hospital; a mortality rate of 12.8%. One thousand three hundred patients (50.1%) had a SOFA score of 2 or more, whilst 886 patients (34.2%) had an increase of 2 points from baseline in the SOFA score. RH was found in 79 patients, 3% of the study population. Baseline variables for the study population are presented in Table 1. None of the continuous baseline data were normally distributed. Missing data: Lactate 197 (7.6%); INR 428 (16.5%) and Albumin 183 (7.1%).

**Table 1.** The baseline characteristics of the study population.

	All (*n* = 2594 Median [IQR] or number (percentages)	Survivors (*n* = 2262) Median [IQR] or number (percentages)	Non-survivors (*n* = 332) Median [IQR] or number (percentages)	Difference between survivors and non-survivors
Patient characteristics				
Age (years)	73 [57–84]	72 [56–83]	80 [72–88]	*p* < .0001
Sex (male)	1380 (53.2%)	1189 (52.6%)	192 (57.8%)	*p* = .08
Respiratory rate (breaths/minute)	23 [19–28]	22 [19–28]	25 [20–32]	*p* < .0001
Oxygen saturation (%)	96 [93–97]	96 [93–98]	94 [89.5–96]	*p* < .0001
Fraction of inspired oxygen (FiO_2_)	0.21 [0.21–0.21]	0.21 [0.21–0.21]	0.21 [0.21–0.28]	*p* < .0001
Heart rate (beats/minute)	102 [87–118]	102 [88–118]	101 [83–118]	*p* = .32
Systolic blood pressure	127 [110–144]	127 [111–145]	119 [102–143.5]	*p* < .0001
Temperature (degrees centigrade)	37.2 [36.6–38.2]	37.3 [36.6–38.3]	36.8 [36.4–37.6]	*p* < .0001
Altered mental state (new)	923 (35.6%)	748 (33.1%)	175 (52.7%)	*p* < .0001
Refractory hypotension	79 (3%)	51 (2.3%)	28 (8.4%)	*p* < .0001
Blood results				
WCC (×10^9^/l)	11.8 [8.2–16]	11.8 [8.2–15.9]	12.0 [8.7–16.3]	*p* = .33
Platelets (×10^9^/l)	250 [188–328]	251 [189–328]	243 [177–325]	*p* = .09
International normalized ratio (INR)	1.2 [1.1–1.4]	1.2 [1.1–1.3]	1.2 [1.1–1.6]	*p* < .0001
Urea (mmol/l)	7.1 [4.8–11.4]	6.7 [4.6–10.1]	12.7 [7.5–20.7]	*p* < .0001
Creatinine (micromol/l)	90 [69–129]	88 [68–122]	115.5 [77–187]	*p* < .0001
Bilirubin (micromol/l)	10 [6–15]	9 [6–15]	10 [7–16]	*p* = .15
Albumin (g/l)	32 [28–36]	32 [28–36]	28 [23–32]	*p* < .0001
C-reactive protein (mg/l)	69 [23–152]	67 [22–146]	92 [33–188]	*p* = .0001
Lactate (mmol/l)	1.6[1.1–2.5]	1.5 [1.1–2.3]	2.2 [1.4–3.9]	*p* < .0001
Co-morbidities				
Dementia	393 (15.2%)	309 (13.7%)	84 (25.3%)	*p* < .0001
Malignancy	395 (15.2%)	340 (15%)	55 (16.7%)	*p* = .46
-with metastases	240 (9.3%)	203 (9%)	37 (11.1%)	*p* = .22
NH residency or live-in carer or minimum TDS care package	729 (28.1%)	568 (25.1%)	161 (48.5%)	*p* < .0001
Long-term oxygen therapy (LTOT)	48 (1.9%)	36 (1.6%)	12 (3.6%)	*p* = .001
Community or previous in-hospital DNAR orders	276 (10.6%)	197 (8.7%)	79 (23.8%)	*p* < .0001
Any of the above co-morbidities	1248 (48.1%)	1015 (44.9%)	233 (70.2%)	*p* < .0001
Source of infection/sepsis				
Respiratory	1065 (41.1%)	872 (38.5%)	193 (58.1%)	*p* < .0001
Urogenital	440 (17%)	416 (18.4%)	24 (7.2%)	*p* < .0001
Abdomen	178 (6.7%)	161 (7.1%)	17 (5.1%)	*p* = .20
Soft tissue	156 (6.0%)	143 (6.3%)	13 (3.9%)	*p* = .11
Ear, nose and throat	36 (1.4%)	36 (1.6%)	0 (0%)	*p* = .01
Central nervous system	21 (0.8%)	19 (0.8%)	2 (0.6%)	*p* = 1.0
Bone	12 (0.5%)	10 (0.4%)	2 (0.6%)	*p* = .66
Device	12 (0.5%)	12 (0.5%)	0 (0%)	*p* = .38
Unknown	311 (12%)	268 (11.8%)	43 (12.9%)	*p* = .02
Not infection	363 (14%)	325 (14.4%)	38 (11.4%)	*p* = .18
Scores				
REDS	2 [2–4]	2 [1–3]	4 [3–5]	*p* < .0001
SIRS	2 [2–3]	2 [2–3]	2 [2–3]	*p* = .74
NEWS2	5 [4–8]	5 [4–7]	8 [5–10]	*p* < .0001
CURB65	2 [1–3]	2 [1–3]	3 [2–4]	*p* < .0001
SOFA	2 [1–3]	1 [0–3]	3 [2–5]	*p* < .0001
MEDS	7 [5–9]	6 [3–8]	10 [7–12]	*p* < .0001
PIRO	8 [5–11]	8 [5–11]	11.5 [9–14]	*p* < .0001
Treatments				
Time to antibiotics (minutes)	102 [55–171.5]	105 [56–172]	82 [50–158]	*p* = .002
Volume of intravenous fluid (ml)	1000 [500–1000]	1000 [500–1000]	1000 [500–1500]	*p* = .16
Admission to the intensive care unit	223 (8.6%)	174 (7.7%)	55 (16.6%)	*p* < .0001
Hospital length of stay (days)	5 [2–12]	5 [2–12]	5 [2–15]	*p* = .96
Arrival by ambulance	2160 (83.3%)	1844 (81.5%)	316 (95.2%)	*p* < .0001

IQR: inter-quartile range; NH: nursing home; DNAR: do not attempt resuscitation; TDS: ter die sumendum (three-times-a-day); REDS: risk-stratification of emergency department suspected sepsis; SIRS: systemic inflammatory response syndrome; NEWS2: national early warning score 2, CURB65: confusion urea respiratory rate blood pressure 65 (years); SOFA: sequential organ failure assessment; MEDS: mortality in emergency department sepsis; PIRO: patient infection response organ.

**Figure 2.**
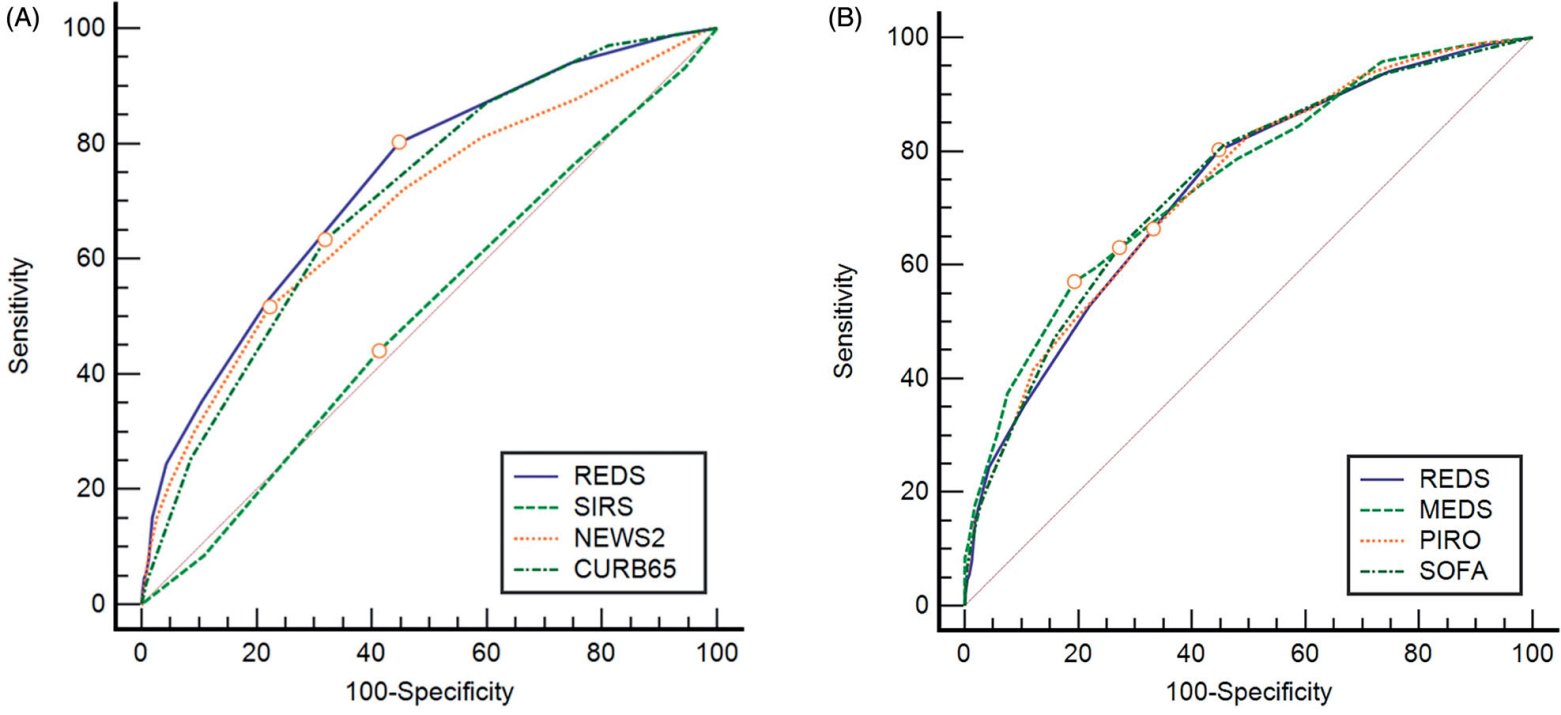
Receiver operator characteristic (ROC) curves for the REDS and comparator scores, for in-hospital mortality. REDS: risk-stratification of emergency department suspected sepsis; SIRS systemic inflammatory response syndrome; NEWS2: national early warning score 2; SOFA: sequential organ failure assessment; MEDS: mortality in emergency department sepsis; PIRO: patient infection response organ; CURB65: confusion urea respiratory rate blood pressure 65 (years); AUROC: area under receiver operator characteristic curve for the REDS score 0.73 (95% confidence interval [CI] 0.72–0.75); SIRS criteria 0.51 (95% CI 0.49–0.53); NEWS2 0.69 (95% CI 0.67–0.70), CURB65 0.71 (95% CI 0.69–0.72); SOFA score 0.74 (95% CI 0.72–0.76), MEDS score 0.75 (95% CI 0.73–0.75); PIRO 0.74 (95% CI 0.72–0.75).

Figure 2(A) illustrates the ROC curves for the REDS score, the SIRS criteria, the NEWS2 and CURB65 scores. Figure 2(B) illustrates the ROC curves for the REDS, SOFA, MEDS and PIRO scores.

The significance of the difference in AUROC between the REDS score and the comparator score and the test characteristics of the high-risk groups in each score studied are presented in Table 2 together with the results of the Hosmer–Lemeshow test. The AUROC curve of the REDS score was significantly greater than that of the SIRS criteria and the NEWS2 scores. The difference in AUROC curves for the REDS score and CURB65 scores did not reach statistical significance in the whole population but did so when patients with missing variables were excluded (Table 3). The AUROC curve of the REDS score is similar to that of the SOFA, MEDS and PIRO scores. The sensitivity for mortality of the high-risk category at the cut-off points identified was greatest for the REDS score at 80.1% (95% confidence interval [CI] 75.4–84.3). This was significantly greater than that for all the comparator scores. The sensitivity for mortality at the cut-off point of ≥8 for the MEDS score of 74.1% (95% CI 69.0–78.7) was similar to that for the REDS score, as the 95% CI_S_ overlap. A NEWS 2 score of ≥3 had the highest sensitivity and lowest specificity for mortality. An increase of 2 points from baseline, in the SOFA score was associated with a sensitivity for mortality of only 62.95% (95% CI 57.5–68.2).

**Table 2.** The AUROC curves, the cut-off points and the test characteristics of the REDS and comparator scores.

Score	H-L test	AUROC (95% CI)	Significance of difference in AUROC curve compared with REDS score	Cut-off score	Sensitivity percentage (95% CI)	Specificity percentage (95% pI)	PPV percentage (95% CI)	NPV percentage (95% CI)	Accuracy percentage (95% CI)
REDS	*p* = .61	0.73 (0.72–0.75)	Not applicable	≥3	80.1 (75.4–84.3)	55.3 (53.2–57.3)	20.8 (19.7–22)	95 (93.8–95.9)	58.4 (56.5–60.4)
				≥5	35.2 (30.1–40.6)	89.5 (88.2–90.8)	33.1 (29.0–37.4)	90.4 (89.7–91.1)	82.6 (81.1–84.0)
				≥7	15.1 (11.4–19.4)	98.1 (97.5–98.7)	54.4 (44.5–63.8)	88.7 (88.3–89.2)	87.5 (86.2–88.8)
SIRS	*p* = .02	0.51 (0.49–0.53)	*p* < .0001	≥2^a^	76.5 (71.6–81.0)	24.7 (23.0–26.5)	13.0 (12.3–13.7)	87.8 (85.4–89.8)	31.3 (29.6–33.2)
NEWS2	*p* = .41	0.69 (0.67–0.70)	*p* = .005	≥8	51.5 (46.0–57.0)	77.8 (76.0–79.5)	25.4 (23.0–28.0)	91.6 (90.7–92.5)	74.4 (72.7–76.1)
				≥7^a^	59.9 (54.4–65.3)	67.7 (65.7–69.6)	21.4 (19.7–23.2)	92.0 (91.0–92.9)	66.7 (64.8–68.5)
				≥5^a^	81.0 (76.4–85.1)	40.9 (38.9–43.0)	16.8 (15.9–17.6)	93.6 (92.1–94.9)	46.0 (44.1–48.0)
				≥3^a^	97.6 (95.3–98.9)	5.8 (4.9–6.8)	13.2 (13.0–13.4)	94.2 (89.0–97.1)	17.5 (16.1–19.1)
				3 in one vital sign^a^	86.1 (82.0–89.9)	31.4 (29.5–33.4)	15.6 (14.9–16.3)	93.9 (92.1–95.3)	38.4 (36.5–40.3)
				≥5 or 3 in one vital sign^a^	91.0 (87.4–93.8)	24.6 (22.9–26.5)	15.1 (14.5–15.6)	94.9 (92.9–96.3)	33.1 (31.3–35.0)
CURB65	*p* = .14	0.71 (0.69–0.72)	*p* = .05	≥3	63.3 (57.8–68.5)	68.1 (66.2–70.0)	22.6 (20.8–24.4)	92.7 (91.6–93.6)	67.5 (65.7–69.3)
SOFA	*p* = .22	0.74 (0.72–0.76)	*p* = .61	≥3	63.0 (57.5–68.2)	72.9 (71.0–74.7)	25.4 (23.4–27.5)	93.1 (92.1–93.9)	71.6 (69.8–73.3)
ΔSOFA	N/A	N/A	N/A	≥2^a^	63.0 (57.5–68.2)	70.1 (68.1–72.0)	23.6 (21.8–25.5)	92.8 (91.8–93.7)	69.2 (67.3–70.9)
MEDS	*p* = .02	0.75 (0.73–0.77)	*p* = .32	≥10	56.9 (51.4–62.3)	80.8 (79.1–82.4)	30.3 (27.7–33.0)	30.3 (27.7–33.0)	77.7 (76.1–79.3)
				≥8^a^	74.1 (69.0–78.7)	58.4 (56.4–60.5)	20.7 (19.5–22.1)	93.9 (92.7–94.9)	60.5 (58.5–62.3)
PIRO	*p* = .3	0.74 (0.72–0.75)	*p* = .85	≥10	66.3 (60.9–71.3)	66.8 (64.8–68.7)	22.7 (21.0–24.4)	93.1 (92.1–94.0)	66.7 (64.9–68.5)

^a^Other cut-off points advocated in the literature.

H-L: Hosmer–Lemeshow; AUROC: area under receiver operator characteristic curve; CI: confidence interval; REDS: risk-stratification of emergency department suspected sepsis; SIRS: systemic inflammatory response syndrome; NEWS2: national early warning score 2; CURB65: confusion urea respiratory rate blood pressure 65 (years); SOFA: sequential organ failure assessment; MEDS: mortality in emergency department sepsis; PIRO: patient infection response organ.

**Table 3.** The AUROC curves, the cut-off points and the test characteristics of the REDS and comparator scores after exclusion of patients with missing variables.

Score	HL test	AUROC (95% CI)	Significance of difference in AUROC curve compared with REDS score	Cut-off Score	Sensitivity percentage (95% CI)	Specificity percentage (95% CI)	PPV percentage (95% CI)	NPV percentage (95% CI)	Accuracy percentage (95% CI)
REDS	*p* = .67	0.74 (0.72–0.76)	N/A	≥3	82.9 (77.9–87.2)	51.8 (49.4–54.2)	21.4 (20.2–22.6)	95.0 (93.6–-96.2)	56.1 (53.8–58.3)
				≥5	38.3 (32.5–44.4)	88.0 (86.3–89.5)	33.4 (29.2–38.0)	90.0 (89.1–90.9)	81.2 (79.4–82.9)
				≥7	16.7 (12.5–21.7)	97.7 (96.8–98.3)	52.9 (42.8–62.8)	88.1 (87.6–88.7)	86.6 (85.0–-88.1)
SIRS	*p* = .02	0.51 (0.48–0.52)	*p* < .0001	≥2^a^	77.0 (71.5–81.9)	22.4 (20.4–24.4)	13.6 (12.8–14.4)	86.0 (82.9–88.6)	29.8 (27.8–31.9)
NEWS2	*p* = .12	0.68 (0.66–0.70)	*p* = .004	≥8	53.5 (47.4–59.6)	75.7 (73.6–77.8)	25.9 (23.3–28.6)	91.2 (90.1–92.2)	72.7 (70.7–74.7)
CURB65	*p* = .33	0.70 (0.68–0.72)	*p* = .049	≥3	64.7 (58.7–70.4)	67.0 (64.7–69.2)	23.6 (21.7–25.7)	92.3 (91.1–93.4)	66.7 (64.5–68.8)
SOFA	*p* = .69	0.74 (0.72–0.76)	*p* = .71	≥3	65.1 (59.0–70.8)	71.0 (68.8–73.1)	2.6.2 (24.0–28.4)	92.8 (91.6–93.8)	70.2 (68.1–72.2)
ΔSOFA	N/A	N/A	N/A	≥2^a^	65.1 (59.0–70.8)	68.0 (65.7–70.2)	24.3 (22.3–26.4)	92.5 (91.3–93.6)	67.6 (65.5–69.6)
MEDS	*p* = .21	0.75 (0.73–0.76)	*p* = .57	≥10	57.6 (51.7–63.6)	79.9 (77.9–81.7)	31.1 (28.2–34.2)	92.3 (91.2–93.2)	76.8 (74.9–78.7)
PIRO	*p* = .33	0.73 (0.71–0.75)	*p* = .84	≥10	66.5 (60.6–72.2)	65.1 (62.8–67.4)	23.2 (21.3–25.1)	92.5 (91.2–93.6)	65.3 (63.2–67.4)

^a^Other cut-off points advocated in the literature.

H-L: Hosmer–Lemeshow; AUROC: area under receiver operator characteristic curve; CI: confidence interval; REDS: risk-stratification of emergency department suspected sepsis; SIRS: systemic inflammatory response syndrome; NEWS2: national early warning score 2; CURB65: confusion urea respiratory rate blood pressure 65 (years); SOFA: sequential organ failure assessment; MEDS: mortality in emergency department sepsis; PIRO: patient infection response organ.

The AUROC for the MEDS score of 0.75 (95% CI 0.73–0.77) was significantly greater than the AUROC curve when the subjective opinion of the doctor was excluded from the score, 0.73 (95% CI 0.71–0.75), *p* < .0001.

Test characteristics for all the scores were re-calculated after excluding 623 patients with missing data. Of this population of 1971 patients, 269 died in hospital, a mortality rate of 13.6%. The test characteristics are presented in Table 3. All scores except the SIRS criteria and the MEDS score in the full dataset, showed satisfactory calibration when the Hosmer–Lemeshow test for goodness-of-fit, was applied.

**Figure 3.**
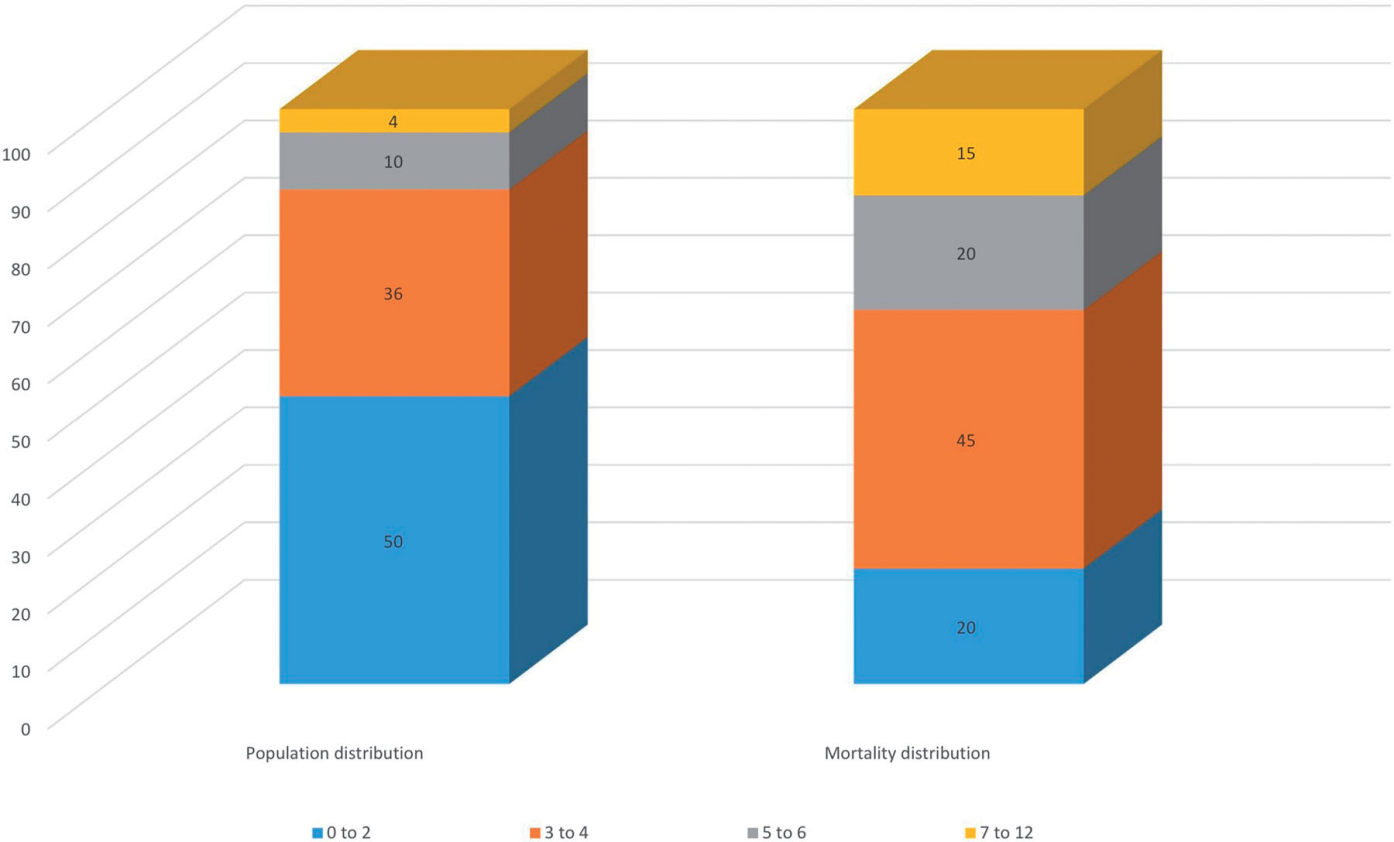
The percentage distribution of the REDS score through the study population and the percentage distribution of the REDS score through the deceased population. REDS score: risk-stratification of emergency department suspected sepsis score. Percentages have been rounded to the closest 1%.

Figure 3 illustrates the population and mortality distribution of the REDS score. The percentages have been rounded to the nearest 1%. This figure shows that 35% of the in-hospital deaths occurred in patients with REDS scores of 5–12 who make-up 14% of the population.

The mortality rates associated with REDS score of 0–2, 3–4, 5–6 and ≥7 were 5%, 16.1%, 25.6% and 54.3%. Re-analysis of the study population after exclusion of the 276 patients with a previous DNAR order (either community or in-hospital) showed that the REDS score had an AUROC curve of 0.73 (95% CI 0.71–0.75) with a cut-off point of ≥3. The mortality rates for those with REDS scores of 0–2, 3–4, 5–6 and ≥7 were 4.5%, 13.7%, 23.5% and 52.1%, respectively.

## Discussion

In this study, we found that the REDS score had greater discrimination for mortality than the SIRS criteria and the NEWS2 scores but equivalent to the other scores studied. It also had a better sensitivity for mortality than the CURB65, SOFA, MEDS and PIRO scores, at their respective cut-off points identified by the statistical software program. The REDS was also more objective than the MEDS score and had fewer variables than the PIRO score. Based on these criteria, the REDS score was better than all the comparator scores to risk-stratify ED suspected sepsis patients, for mortality.

The population we studied were elderly (median age 73 years) and infirm with significant co-morbidities. In fact, 48% of our study population and 70% of those who died had at least one of the following co-morbidities; dementia, malignancy and inability to live independently were on LTOT or had a previous DNAR decision. The overall mortality rate was 12.8%, 83% arrived by ambulance and only 8.6% of the population were admitted to the ICU. But this demographic is not unusual. In a recent study of over 500 ED patients admitted with suspected sepsis, Sabir et al. [34] reported a median age of 74 years and an overall mortality rate of 13.2%. In their study too, 80% arrived by ambulance and only 6.5% were referred to ICU. Similarly, a study of 2043 patients by Abdullah et al. [17] in Denmark, showed the median age to be 73.2 (IQR 60.9–82.1) years. They too found that only 7.5% of their population were transferred to the ICU. Furthermore, only 1% of their population received vasopressors. In our study population, only 3% had RH. Many of these patients may not have been candidates for intensive care treatment and thus initiation of vasopressors.

We found that the SIRS criteria did not calibrate nor discriminate for mortality with an AUROC curve of 0.51 (95% CI 0.49–0.53). This is similar to the AUROC curve of 0.52 (95% CI 0.47–0.56) found by Abdullah et al. [17] and 0.53 (95% CI 0.49–0.57) found by Zhang et al. [35]. The sensitivity for mortality of two or more SIRS criteria was 76.5% (95% CI 71.6–81.0). This is similar to the 77.2% sensitivity for 30 d mortality found by Brink et al. [36] in their study of 8204 ED patients.

The NEWS 2 score had an AUROC curve 0.69 (95% CI 0.67–0.70) that was significantly less than the REDS score, *p* = .005. The cut-off point of ≥8 determined by the software program identified significantly fewer deaths (1.5% [95% CI 46.0–57.0]) than the high-risk group of the REDS score but with greater specificity (77.8 [95% CI 76.0–79.5]). These test characteristics are not useful in the clinical setting as the sensitivity is too poor to be used for screening and the specificity is too low to implement an intervention. Hence, we explored the other cut-off points that have been advocated in the literature [18–21,29,30]. The most sensitive cut-off point for mortality and better than two or more SIRS criteria, was a NEWS2 score of 3 or more. This cut-off point had a sensitivity for mortality of 97.6% (95% CI 95.3–98.9) and specificity of 5.8% (95% CI 4.9–6.8). Although this high sensitivity may be due to the fact that we used a NEWS 2 score ≥3 as an entry criteria for the vast majority of patients, we did see the same results in the 1078 cases that were used to derive the REDS score (unpublished data). The entry criteria for that dataset were based on the expanded SIRS criteria which included altered mental state and a random glucose of >7.7 mmol/l in the absence of diabetes. We recommend using a NEWS 2 score of ≥3 as a screening tool to identify patients who may benefit from early investigations for suspected sepsis. This role is most suited to this score which does not require blood results.

The CURB65 score [22] had an AUROC curve which was similar to the REDS score (*p* = .05) but had a significantly reduced sensitivity for mortality of 63.3% (95% CI 57.8–68.5) at a cut-off point of ≥3. In addition, the specificity was only 68.1%. As stated above, these test characteristics are not useful in the clinical setting. However, it must be acknowledged that the CURB65 score was designed to risk-stratify pneumonia and not general sepsis. Hence, its underperformance may be explained by its application outside its intended use.

The AUROC curve for the SOFA score of 0.74 (95% CI 0.72–0.76) was similar to that for the REDS score and similar to the AUROC of 0.75 (95% CI 0.68–0.83) found by Jones AE et al. [37]. The sensitivity for mortality of an increase of 2 or more points of 63% (95% CI 57.5–68.2) was similar to the sensitivity for mortality of 67.5% (95% CI 55.9–77.8) found by Abdullah et al. [17].

The MEDS and PIRO scores had AUROC curves that were similar to the REDS score. The sensitivity for mortality above the cut-off point was the greatest for the REDS scores. The cut-off point of 10 that we identified for the MEDS score was higher than the cut-off point of 8 found by Shapiro et al. [25] for the moderate-risk group. Re-analysis of a cut-off point of 8 found the sensitivity for mortality to be higher at 74.1% (95% CI 69.0–78.7).

Our data determined the cut-off point for the PIRO score to be ≥10, similar to that found by Howell et al. [26]. At this cut-off point, we found a sensitivity of 66.3 (95% CI 60.9–71.3) compared to 75 (69.1–80.3) obtained by Howell et al. The PIRO score however, uses 15 variables compared to the eight variables used to calculate the REDS score. The reduced number of variables makes the REDS score easier to calculate. Like the MEDS score, the PIRO score includes nursing home residency as a variable. For previously mentioned reasons inclusion of this variable defeats the purpose of the score.

A REDS score of ≥3 has a sensitivity for mortality of 80.1% (95% CI 75.4–84.3) but the specificity is 55.3% (95% CI 53.2–57.3). The specificity for mortality improves significantly to 89.5% for REDS scores of ≥5 and to 98.1% for scores of ≥7. The nature of the interventions to be implemented should therefore reflect these specificities. All patients with a REDS score of ≥3 are at high risk of deterioration and death and should have a Treatment Escalation Plan (TEP) completed in the ED which can be enacted should the patient deteriorate on the ward. The TEP, a quick-reference document, will hold the information on the level of care and the nature of interventions appropriate for the patient should they deteriorate. The REDS score needs to be externally validated. Its role as a monitoring tool should also be studied.

Patients with REDS scores of 3–4 make up 35% of the population (Figure 3) and have an overall mortality rate of 16.1% (95% CI 14.4–17.99). They also account for 45% of deaths. For patients with REDS scores of 5–6 the stakes are much higher; they make up 10% of the population and have a mortality rate of 25.6%. They account for 20% of the deaths. Patients with REDS scores of ≥7 make up 4% of the population, have a 54.4% mortality rate and are responsible for 15% of the deaths (Figure 3). Together, patients with REDS scores of 5–12 make up 14% of the population but 35% of deaths with a mortality rate of 33%. With such test characteristic, this group deserves further study regarding treatments and care pathways to be implemented in the ED.

At present there are no specific treatments for patients with scores of 3 or more, except for treatment of organ dysfunction, RH [6,13] and lactate clearance [38]. Use of the REDS score will help define groups of patients within the heterogeneous population of patients with suspected sepsis, for further study of treatments.

### Limitation

First, this is a retrospective single-centre study and as such is open to bias and not generalizable. We have attempted to mitigate any bias by including a large dataset. The results will need to be externally validated. Second, the calculation of the MEDS score may be inaccurate as it was calculated retrospectively from the documented clinical notes. This inaccuracy will primarily apply to the clinician’s opinion on the patient’s expected mortality. In addition, we did not score the percentage of band in the neutrophil count as this is not available in our hospital. This may have led to the underestimation of both the MEDS and PIRO scores. Third, the SOFA score may be under-estimated as the patients’ physiology may not have reached the maximum possible whilst in the ED. Fourth, we only studied patients who met the inclusion criteria. There may be patients who died who did not meet the inclusion criteria and will be missed by our ED pathway. Fifth, missing variables were scored zero, so that the entire case was not excluded during the analysis. We took this approach as opposed using imputed values so that it reflected real-life. We also re-analysed the data after excluding patients with missing variables, knowing that the sample-size for this re-evaluation was large enough although this may have led to bias as only sicker patients would have had all the blood tests taken. This is reflected in the slightly higher mortality rate of 13.6% compared to 12.8% for the whole study population. Finally, the re-analysis of the study population after exclusion of those with previous DNAR order may not have excluded all patients who are not for escalation of treatment. This will need to be analysed in a population of patients who are actively considered for full escalation of treatment in a prospective study.

## Conclusion

In this single-centre study, the REDS score had a greater AUROC curve compared to the SIRS criteria and NEWS2 score. Although, the AUROC of the REDS score was similar CURB65, SOFA, MEDS and PIRO scores, the sensitivity for mortality at the cut-off points identified was greatest for the REDS score. This makes the REDS score a better risk-stratification tool. A NEWS2 score of ≥3 had the greatest sensitivity (97.6%) and lowest specificity (5.6%) for mortality. The lack of need for blood tests to calculate the NEWS2 score makes it ideal for use as a screening tool.

### Ethics approval

This study of routinely collected anonymized data did not include an intervention and did not change the normal process of care. It is a retrospective observational study from a single centre. The REDS score was derived and validated in our department and is yet to be externally validated. This study is also from the same department. Until the REDS score is externally validated, it is not generalizable. Such studies, in accordance with national Health Research Authority guidance do not require formal ethics approval [39]. In accordance the National Health Research Authority guidance around the General Data Protection Regulation and the Data Protection Act of 2018, patient consent is not required for the analysis of anonymized data [40]. This study has been registered with the Clinical Effectiveness and audit office of St George’s University Hospital as a service evaluation study, under the registration code AUDI000876.

### Consent for publication

Not applicable.

## Data Availability

All data used is included in the manuscript.
